# Potentiation of immune checkpoint blockade with an ITPP radiosensitizer studied with oxygen saturation measurements from photoacoustic imaging

**DOI:** 10.1038/s41598-025-05930-0

**Published:** 2025-07-01

**Authors:** Renee L. Tran, Xiaofei Liang, Jorge de la Cerda, F. William Schuler, Mark D. Pagel

**Affiliations:** 1 Department of Cancer Systems Imaging, MD Anderson Cancer Center, 1515 Holcombe Blvd., Houston, TX 77030 USA; 2 University of Texas Graduate School of Biomedical Sciences, Houston, TX USA; 3 Department of Medical Physics, University of Wisconsin, Madison, WI 53705 USA

**Keywords:** Tumor hypoxia, ITPP, Immune checkpoint blockade, Photoacoustic imaging, Cancer imaging, Molecular imaging

## Abstract

Hypoxia in the tumor microenvironment hinders antitumor immunity. Increasing tumor oxygenation may promote T cell infiltration and tumor control by immune checkpoint blockade (ICB). We found that a radiosensitizer, myo-inositol trispyrophosphate (ITPP), caused oxygen unloading from hemoglobin in CT26 and 4T1 tumors as indicated by photoacoustic imaging (PAI). This change in hypoxia detected by PAI was correlated with strong positive correlations with CD8+ and CD4+ FoxP3- effector T cell (Teff), and negative correlations with monocyte frequencies, indicating that ITPP promoted more immunogenic tumor microenvironments in both models. Combination ITPP and ICB improved tumor control and survival in both models. Therefore, imaging ITPP-modulated tumor hypoxia with PAI was related to ICB treatment response in these studies. Future combination immunotherapy regimens may benefit from monitoring hypoxia using molecular imaging with PAI.

## Introduction

Immune checkpoint blockade (ICB) treatments have joined surgery, chemotherapy, and radiation therapy as the fourth pillar of cancer treatment^1^. ICB was first approved for patients with advanced melanoma, and ICB is now approved for the treatment of patients with lung lung, prostate, ovarian, cervical, endometrial, and urothelial cancers, as well as hepatocellular and renal cell carcinomas^2–10^. However, ICB is still far from being a reliable cure for cancer. For example, the ICB treatments nivolumab and ipilimumab (the therapeutic antibodies anti-programmed death-1, or αPD-1, and anti-cytotoxic T lymphocyte antigen-4, or αCTLA-4, respectively) induce objective response rates of 58% in melanoma^11^, 35.9% in non-small cell lung cancer^12^, 42% in renal cell carcinoma^13^, 16% in hepatocellular carcinoma^14^, and 18% in breast cancer^15^. To increase the efficacy of ICB, more studies are needed to sensitize tumors and identify patients who would be the most responsive to ICB.

Hypoxia in the tumor microenvironment (TME) can contribute to resistance to ICB^16^. Tumor hypoxia recruits and promotes the immunosuppressive function of myeloid-derived suppressor cells (MDSCs)^17,18^. In addition, hypoxia increases the stability and transcriptional activity of hypoxia-inducible factor HIF-1α^19^, which subsequently upregulates programmed death ligand-1 (PD-L1) that increases the inhibitory landscape in hypoxic TMEs^20,21^. MDSCs and the increased PD-L1 expression recruited and induced by hypoxia promote the development of immunogenically “cold” tumors by inhibiting T cell function. Hypoxia also causes tumors to become “cold” by directly preventing T cell trafficking into hypoxic regions, inhibiting T cell expansion, inhibiting tumor cell cytotoxicity, and promoting T cell death^22–24^. Efforts have focused on converting “cold” tumors into “hot” tumors that have high T cell penetrance and activity and causing such tumors to respond better to ICB^1,25^. Studies targeting hypoxic regions using hypoxia-activated prodrugs^22^, inhibitors of oxidative phosphorylation^26,27^, agents for direct oxygenation^28^, and oxygen carriers^29^, all showed that doing so promotes T cell infiltration and enhances ICB response. However, the extent of hypoxia abrogation necessary to increase ICB efficacy is unclear.

Myo-inositol trispyrophosphate (ITPP) is an allosteric effector that increases oxygen unloading by red blood cells (RBCs)^30^, leading to increased oxygenation in tumors^31–35^. Furthermore, ITPP may contribute to producing a “hot” tumor by increasing immune cell influx, reducing the frequencies of myeloid and regulatory T cell populations in tumors, and decreasing PD-L1 expression in the tumor^34–36^. A recent clinical trial (NCT02528526) assessing tumor control by ITPP found that 52% of patients treated with ITPP had stable disease and 60% of patients treated with ITPP and subsequent chemotherapy had stable disease^37^. Preclinical studies have tested ITPP as a monotherapy^32,36–42^, and in combination chemotherapy^34,43^ and radiotherapy^31,35,44^ with mixed results. Surprisingly, ITPP has not been tested in combination with ICB such as αPD-1 and αCTLA-4^45,46^. In this study, we take the paradigm of combination therapy with ITPP a step further by postulating that ITPP potentiates ICB tumor response.

To investigate this next-step paradigm, we investigated non-invasive imaging as a tool to monitor HbO_2_ in pre-clinical tumor models. Other studies have used EPR imaging to monitor pO2 in the extracellular tumor microenvironment^31–33^, MRI to indirectly evaluate oxygenation through water relaxation^32,47^, and Bioluminescence imaging and PET to qualitatively assess intracellular hypoxia^35,47^. We elected to use photoacoustic imaging (PAI; also known as multispectral optoacoustic tomography) as a more direct interrogation of the effect of ITPP on HbO_2_^48,49^. PAI can measure the relative concentrations of deoxyhemoglobin (Hb) and oxyhemoglobin (HbO_2_) based on their signature PA spectral profiles after absorbance of near-infrared light^50,51^. These measurements can be used determine % saturation of oxygen (%sO_2_ = HbO_2_/(HbO_2_ + Hb)), which should decrease when ITPP increases oxygen unloading that converts HbO_2_ into Hb. Another study used PAI to evaluate the effects of ITPP as a pre-clinical tumor treatment, but did not report %sO_2_ values in synchrony with ITPP treatment^36^. In this study, we postulated that non-invasive tracking of %sO_2_ with PAI in synchrony with ITPP treatment can improve our understanding how ITPP may potentiate ICB response.

## Results

### ITPP sensitized CT26 tumors to ICB

#### ITPP promoted oxygen unloading in CT26 tumors

We chose CT26 colon carcinoma as our murine model for this test, based in its relative susceptibility to ICB and its well-characterized hypoxic TME^52–54^. Furthermore, CT26 was developed in the albino Balb/cJ background, which is imperative for our studies as PAI is sensitive to melanin.

**Fig. 1:**
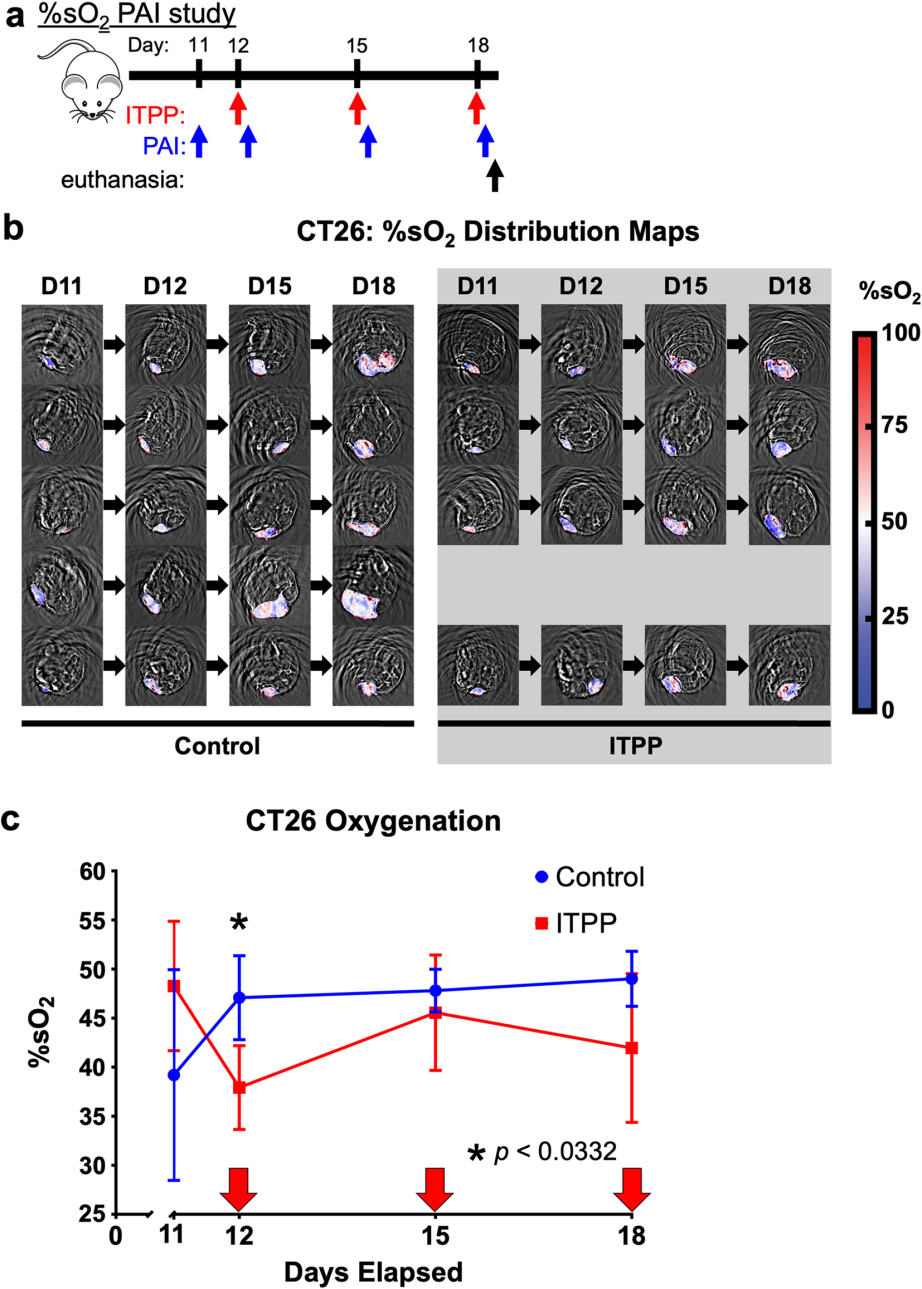
ITPP increased oxygen delivery to CT26 tumors. (**a**) A study timeline. PAI was performed 3 h after administering ITPP. Mice were euthanized after the PAI scan. (**b**) The %sO_2_ distribution maps of CT26 tumors subcutaneously implanted in female Balb/cJ mice, subdivided by treatment. (**c**) The change in %sO_2_ throughout the treatment period of the CT26 model receiving PBS control or ITPP treatment. Arrows indicate days when mice received treatment. Values show the average tumor %sO_2_ and error bars represent standard deviation. *p < 0.0332, *n* = 4–5, field of view 25 × 25 mm.

We tracked the effect of three longitudinal doses of ITPP on %sO_2_ to indirectly monitor a decrease in tumor hypoxia and whether the change is durable. PAI was performed three hours after administration of ITPP or PBS as control on Days 12, 15, and 18 (relative to tumor implantation on Day 0), with the comparative pre-ITPP baseline PAI scan taken on Day 11 (Fig. 1a,b). Our results showed a marked decrease in %sO_2_ three hours after the first dose of ITPP (Fig. 1c), which reflects oxygen unloading that converts HbO_2_ into Hb. This decrease in %sO_2_ observed with the first dose of ITPP was not seen with the second or third doses of ITPP, demonstrating that the oxygen unloading by ITPP was not durable in our study.

#### Oxygen unloading caused by ITPP correlated with increased Teff and decreased monocyte frequencies in CT26 tumors

**Fig. 2:**
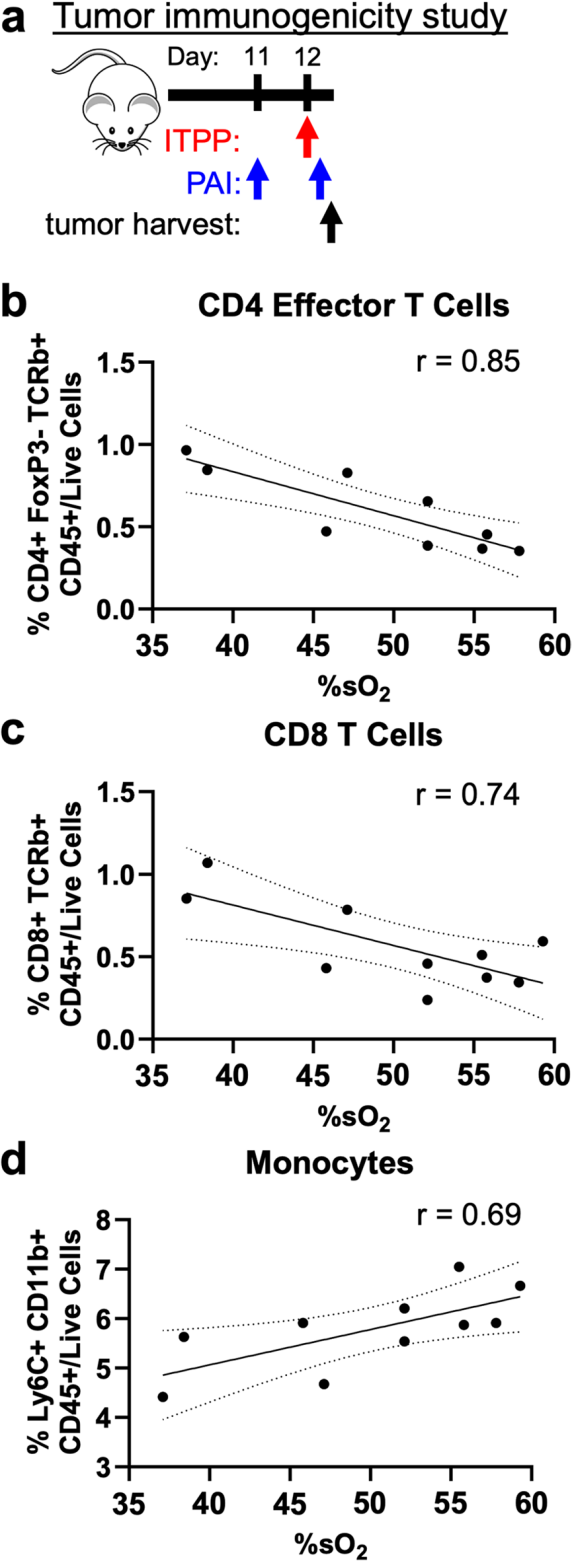
ITPP improved CT26 tumor immunogenicity. (**a**) A study timeline. ITPP was administered on Day 12, then PAI was performed 3 h later, and then immediately followed by tumor harvest for cell analysis. The correlation between the frequencies of %sO_2_ and (**b**) CD4 + FoxP3-TCRb + CD45 + /live cells, (**c**) CD8 + TCRb + CD45 + /live cells, or (**d**) Ly6C + CD11b + CD45 + /live cells in CT26 tumors three hours after the ITPP treatment on Day 12. Dotted lines represent the 95% confidence interval while the solid lines represent the linear best fit line. *n* = 9–10.

Because our previous experiment showed a significant decrease in %sO_2_ three hours after one dose of ITPP, we were particularly interested in whether this change in %sO_2_ was related to the immunogenicity of the tumor as primed by ITPP. To that end, we harvested tumors following PAI that occurred three hours after the first ITPP injection and assessed the immune cell populations by flow cytometry (Fig. 2a). A comparison of the frequencies of immune cell populations in CT26 tumors and %sO_2_ after the first ITPP injection showed that ITPP caused tumors to be more immunogenic, or “hot”. At lower %sO_2_, where oxygen unloading into the tumor TME was greater, we observed greater frequencies of CD8 and CD4 + FoxP3- effector T cells (Teffs) in CT26 tumors (Fig. 2b,c). We also observed a weak correlation between %sO_2_ and the frequencies of Ly6C + monocytic myeloid cells (Fig. 2d). Therefore, ITPP primes CT26 tumors to be “hot” for ICB therapy by increasing Teff while decreasing monocytic population frequencies.

#### Combination ITPP and ICB delayed growth of CT26 tumors

**Fig. 3:**
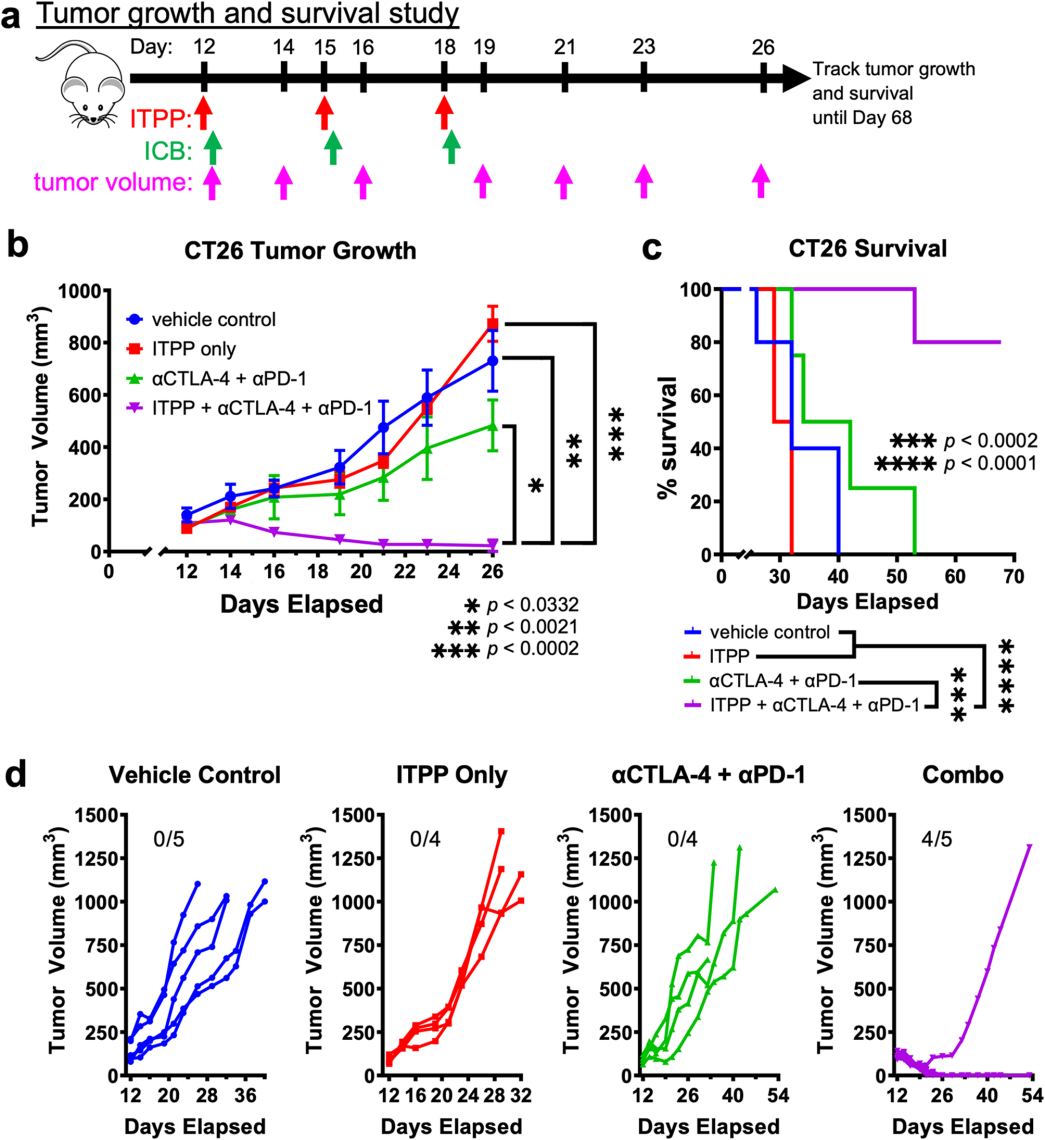
ITPP promoted ICB rejection of CT26 tumors. (**a**) A study timeline. ICB was administered 3 h after ITPP. (**b**) Tumor growth kinetics and (**c**) survival data of Balb/cJ mice bearing subcutaneously implanted CT26 tumors and receiving the indicated treatments. *p < 0.0332, **p < 0.0021, ***p < 0.0002, ****p < 0.0001. (**d**) Individual tumor growth kinetics within each respective treatment group showing the number of tumors rejected. Values are shown as average, and error bars represent standard error of the mean. *n* = 4–5, with three biological repeats.

Because ITPP relieves hypoxia and converts CT26 tumors into “hot” immunogenic tumors three hours after the first ITPP injection, we next asked whether the effect of ITPP to create an immunogenically “hot” TME is sufficient to strengthen the ICB response and promote ICB tumor control. As CT26 tumors are more susceptible to ICB therapy^52–54^, we allowed CT26 tumors to develop further in diameter to 5–6 mm to enhance tumor resistance to ICB. We treated mice bearing the CT26 tumor model with ITPP followed 3 h later with αCTLA-4 and αPD-1 ICB and performed in 3-day intervals (Fig. 3a). We also tested ITPP only, αCTLA-4 and αPD-1 only, and vehicle as controls in the same 3-day intervals. We monitored the treatment responses by measuring tumor volume and tracking mouse survival.

When compared to the three controls, combination ITPP, αCTLA-4, and αPD-1 significantly reduced CT26 tumor growth and increased survival in this treatment group (Fig. 3b,c). The majority of mice receiving combination treatment rejected their tumors while control treatments showed no tumor rejection (Fig. 3d). While this data shows that ITPP promotes ICB tumor control, it should be noted that ITPP significantly decreased %sO_2_ only after the first treatment. Therefore, the initial hypoxic state of the TME may be more consequential in determining ICB tumor response rather than subsequent tumor hypoxic states.

### ITPP sensitized 4T1 tumors to ICB

#### ITPP promoted oxygen unloading in 4T1 tumors

Despite the efficacy of ITPP and ICB in the CT26 model, reproducing these results in a more ICB resistant tumor model is necessary to validate our findings. We chose the 4T1 model as it is less susceptible to ICB compared to the CT26 model, which we also developed in the Balb/cJ background^55,56^.

**Fig. 4:**
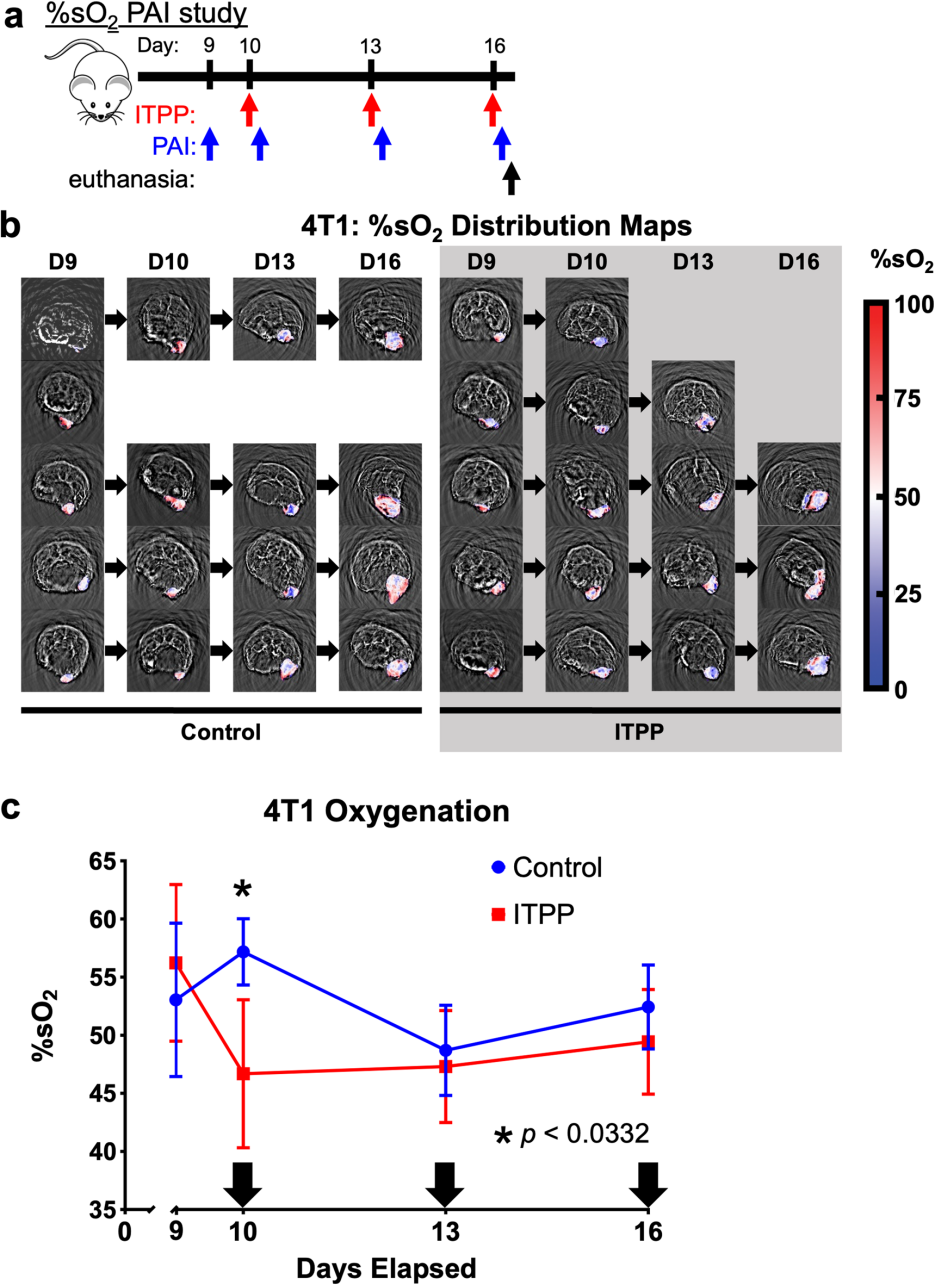
ITPP increased oxygen delivery to 4T1 tumors. (**a**) A study timeline. PAI was performed 3 h after administering ITPP. Mice were euthanized after the last PAI scan. (**b**) The %sO_2_ distribution maps of 4T1 tumors subcutaneously implanted in female Balb/cJ mice, subdivided by treatment. (**c**) The change in %sO_2_ throughout the treatment period of the 4T1 model receiving PBS control or ITPP treatment. Arrows indicate days when mice received treatment. Values show the average tumor %sO_2_ and error bars represent standard deviation. *p < 0.0332, *n* = 4–5, field of view 25 × 25 mm.

We began by assessing oxygen unloading by ITPP in the 4T1 tumors using PAI. Mice bearing orthotopically implanted 4T1 tumors were treated with either ITPP or PBS as control on days 10, 13, and 16, and PAI was conducted on day 9 and three hours after each ITPP treatment (Fig. 4a,b). In a similar manner to the CT26 tumors, ITPP significantly reduced %sO_2_ in 4T1 tumors three hours after the first ITPP treatment (Fig. 4c). Unlike the results with CT26 tumors, continued ITPP treatment showed a slow and gradual recovery of %sO_2_ in the 4T1 tumors. However, there was no significant difference in tumor %sO_2_ between the mice receiving PBS after the second and third injection. Based on these results, ITPP demonstrated the capacity to increase oxygen unloading in 4T1 tumors three hours after the first ITPP treatment.

#### Oxygen unloading correlated with increased Teff and decreased monocyte frequencies in 4T1 tumors

**Fig. 5:**
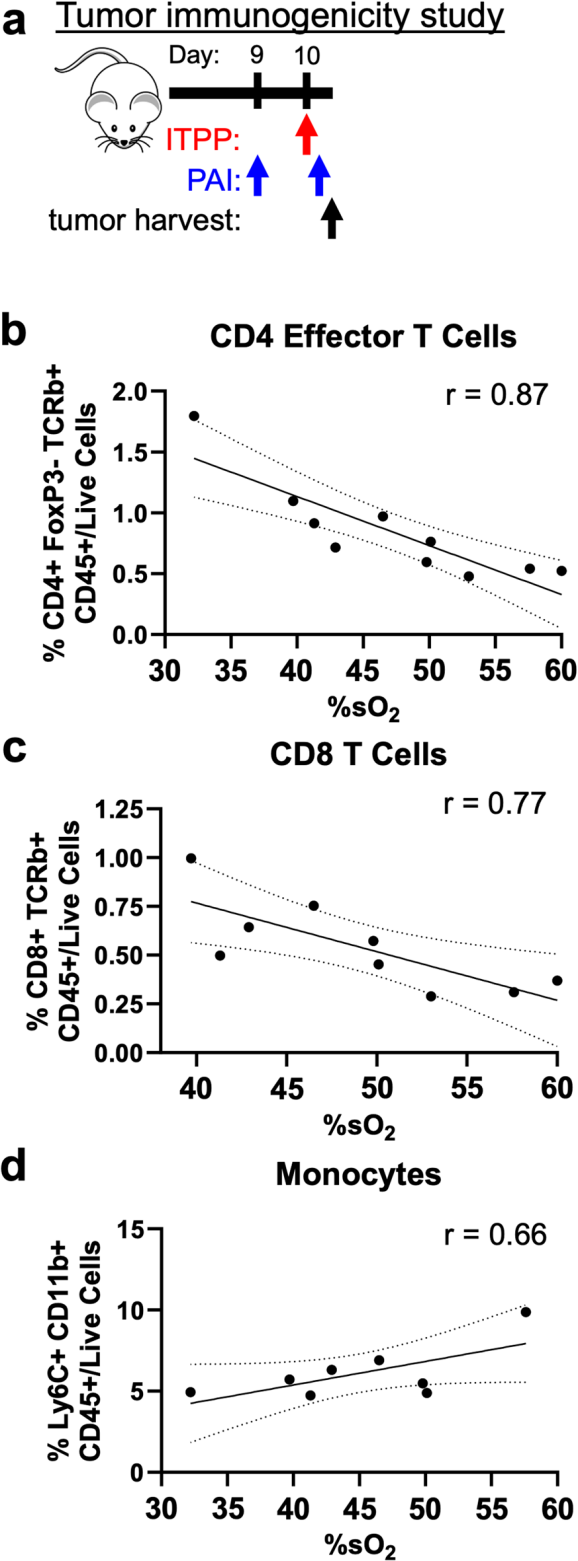
ITPP improved 4T1 tumor immunogenicity. (**a**) A study timeline. ITPP was administered on Day 10, then PAI was performed 3 h later, and then immediately followed by tumor harvest for cell analysis. The correlation between the frequencies of %sO_2_ and A) CD4 + FoxP3-TCRb + CD45 + /live cells, (**b**) CD8 + TCRb + CD45 + /live cells, or (**c**) Ly6C + CD11b + CD45 + /live cells in 4T1 tumors three hours after the ITPP treatment on Day 10. Dotted lines represent the 95% confidence interval while the solid lines represent the linear best fit line. *n* = 8–10.

To assess the efficacy of ITPP for promoting “hot” 4T1 tumors, we harvested 4T1 tumors after PAI three hours following initial ITPP treatment (Fig. 5a). We focused on this timepoint as it was the only timepoint to significantly reduce %sO_2_ in the previous experiment (Fig. 4c). Harvested tumors were then processed using flow cytometry to determine the frequencies of immune cell populations in the tumors.

Results from the flow cytometry analysis correlated similarly to those of CT26 tumors. The frequencies of Teff cells in 4T1 tumors increased with lower %sO_2_ (Fig. 5b,c). Despite correlating weakly, the frequencies of monocytes in 4T1 tumors also increased at higher %sO_2_ (Fig. 5d). As decreased %sO_2_ reflects oxygen unloading in tumor vessels, this data indicates that improved oxygenation in the TME increased Teff infiltration and reduced the presence of immunosuppressive monocytes populations in 4T1 tumors, causing the tumors to become immunogenically “hot”.

#### Combination ITPP and ICB delayed growth of 4T1 tumors

**Fig. 6:**
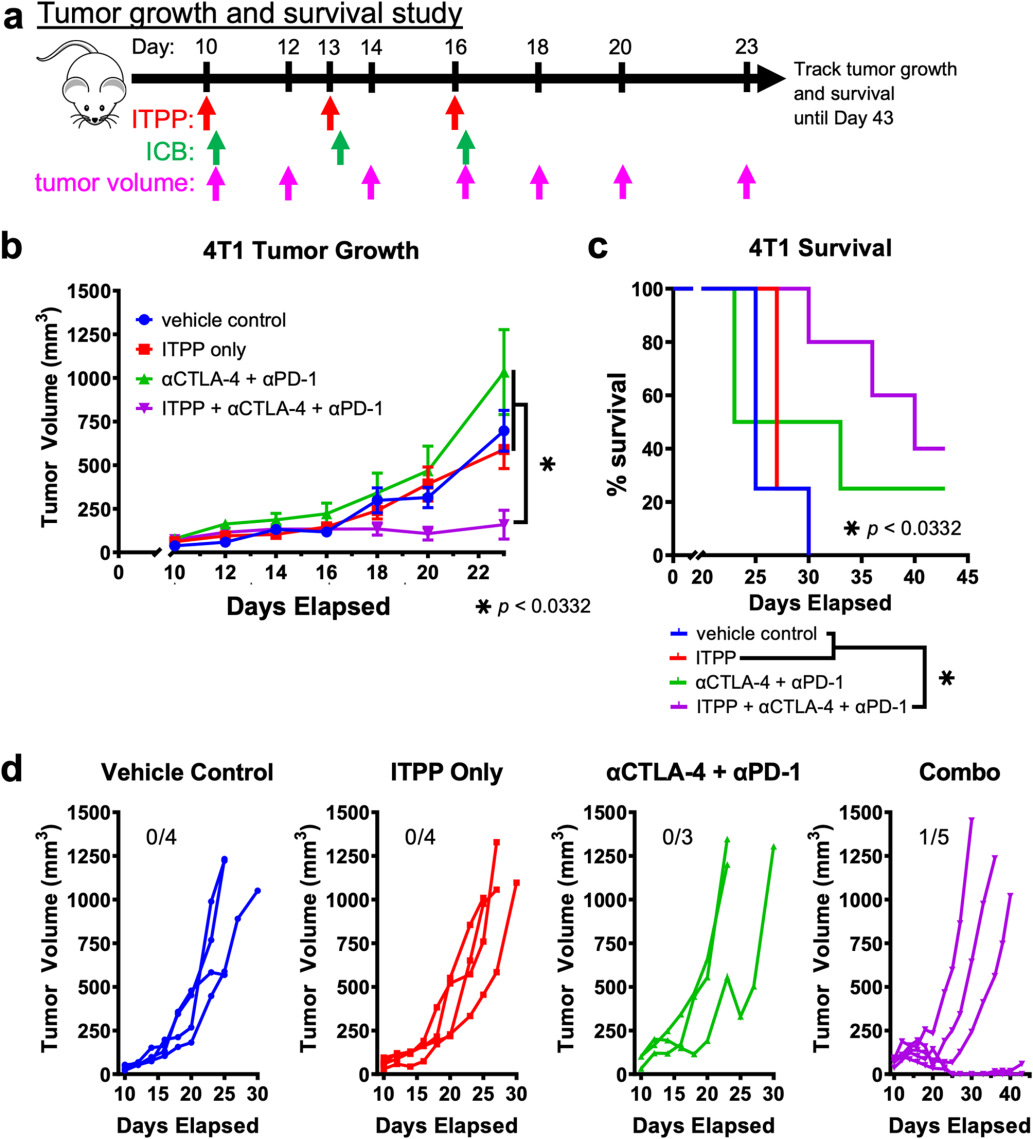
ITPP promoted ICB control of 4T1 tumors. (**a**) A study timeline. ICB was administered 3 h after ITPP. (**b**) Tumor growth kinetics and (**c**) pooled survival data of Balb/cJ mice bearing orthotopically implanted 4T1 tumors and receiving the indicated treatments. (**d**) Individual tumor growth kinetics within each respective treatment group showing the number of tumors rejected. Values are shown as average, and error bars represent standard error of the mean. *p < 0.0332. *n* = 4–5, with three biological repeats.

Similar to our studies with the CT26 model, we assessed whether the immunogenicity induced by ITPP treatment of 4T1 tumors results in enhanced ICB tumor response. We conducted tumor growth studies combining ITPP and followed 3 h later with αCTLA-4 and αPD-1 and repeated in 3-day intervals. We also tested ITPP only, αCTLA-4 and αPD-1 only, and vehicle as controls (Fig. 6a). We measured tumor and mouse survival as indicators of tumor control. Because previous studies found 4T1 tumors to be more resistant to ICB^55,56^, 4T1 tumors were allowed to reach a smaller diameter before initiating treatment (3–4 mm) compared to that of CT26 tumors. Furthermore, we treated 4T1 tumors with four doses of ITPP and/or αCTLA-4 and αPD-1, or vehicle, in three-day intervals, to emphasize the effect of combination ITPP and ICB.

Similar to the results of ITPP and ICB on the CT26 tumor model, the combination of ITPP with αCTLA-4 and αPD-1 significantly delayed 4T1 tumor growth compared to controls (Fig. 6b). The combination treatment also improved survival of mice bearing 4T1 tumors, although this improved survival was not statistically significant when comparing ITPP and ICB vs. ICB alone (Fig. 6c). This improved survival was partly due to a small increase in the frequency of 4T1 tumor rejection (Fig. 6d). For mice to reject some 4T1 tumors after treatment with αCTLA-4 and αPD-1, but not in CT26 tumors, may reflect a difference in tumor susceptibility to ICB for the two models at their respective starting diameters.

### The %sO_2_ indicated tumor control mediated by combination ITPP and ICB by enhancing frequencies of Teffs and Teff subsets

#### The %sO_2_ of tumors after initial ITPP treatment correlated with combination ITPP and ICB tumor response in CT26 and 4T1 tumors

**Fig. 7:**
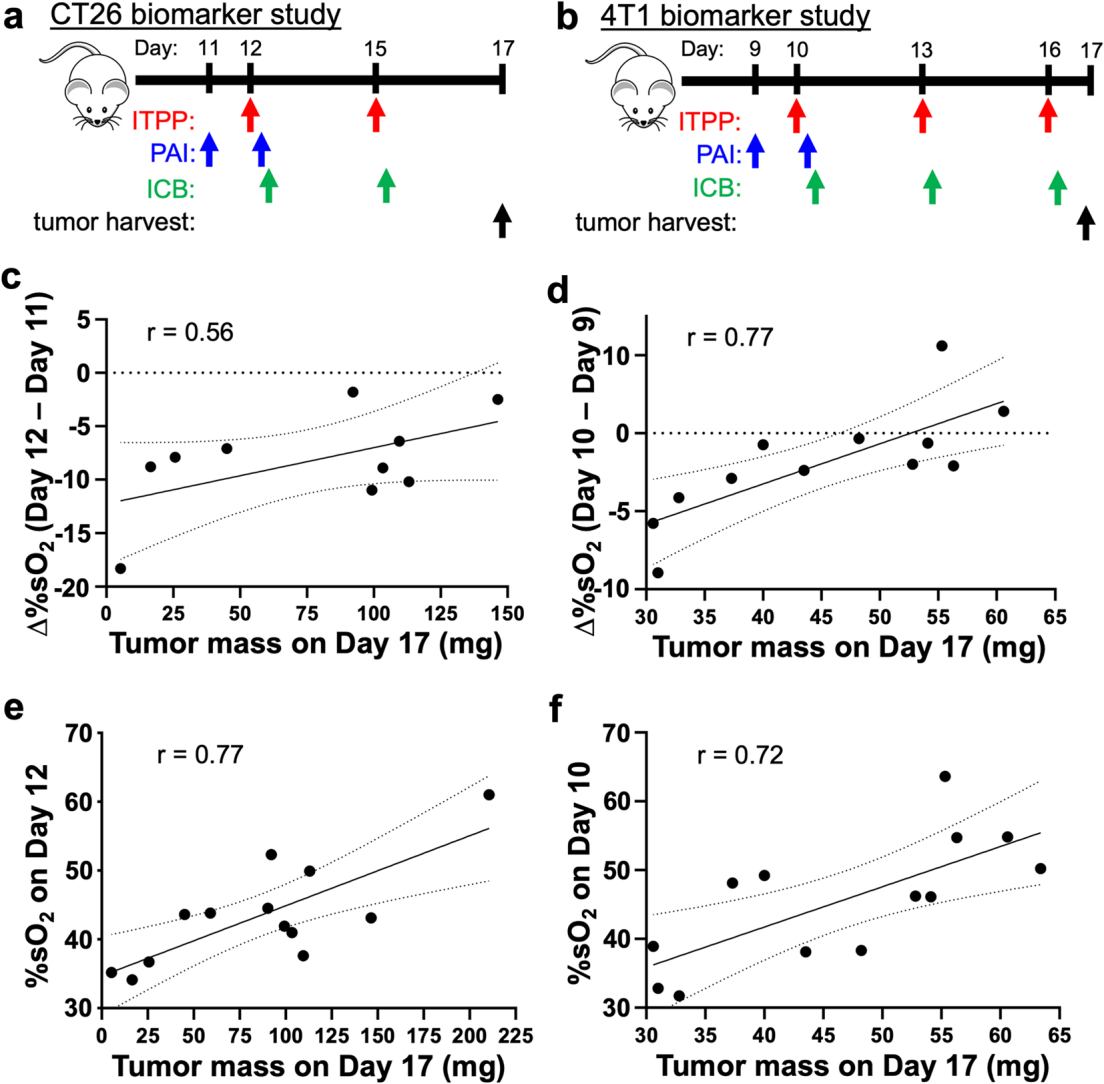
%sO_2_ after the first ITPP treatment positively correlated with combination ITPP and ICB tumor control. (**a,b**) The study timelines. PAI was performed 3 h after administering ITPP. ICB was administered immediately after PAI on the initial treatment day. ICB was administered 3 h after ITPP on days without PAI. The comparison between the change in %sO_2_ (Δ%sO_2_) three hours after initial ITPP treatment relative to pretreatment, compared to the mass of **c**) CT26 and (**d**) 4T1 tumors after two combination ITPP, αCTLA-4 and αPD-1 treatments. A similar comparison between %sO_2_ three hours after initial ITPP treatment and the mass of (**e**) CT26 and **f**) 4T1 tumors after two combination treatments. Dotted lines represent the 95% confidence interval while the solid lines represent the linear best fit line. *n* = 10–13*.*

Because our previous experiments showed that %sO_2_ measurements from PAI (Figs. 1 and 4) indicate that ITPP can prime TMEs to be immunogenically “hot” (Figs. 2 and 5), and that ITPP can potentiate ICB tumor control (Figs. 3 and 6), we postulated that PAI measurements on the day of initiating treatment are related to tumor control after ITPP and ICB at a later day. We were also interested in examining whether Δ%sO_2_ is related to tumor control at a later day. The timeline of this study for both tumor models is shown in Fig. 7a,b.

All of the CT26 tumors and the majority of 4T1 tumors had Δ%sO_2_ values less than 0, indicating that ITPP decreased %sO_2_ and thus promoted oxygen unloading (Fig. 7c,d). The Δ%sO_2_ of both tumor models bracketing initial treatment was correlated with tumor mass 5 days later. Similarly, post-ITPP %sO_2_ in both CT26 and 4T1 tumors was positively correlated with tumor mass after ICB treatment (Fig. 7e,f). The Δ%sO_2_ value is based on two %sO_2_ measurements, and therefore may have more experimental variability, which may explain the difference in correlations between Fig. 7c and d. Most importantly, these results indicated that Δ%sO_2_ and %sO_2_ measured at the start of treatment are early response biomarkers for our specific studies on the day of treatment that foreshadow eventual tumor control on later days.

#### %sO_2_ after initial ITPP treatment correlates with frequencies of Teffs

**Fig. 8:**
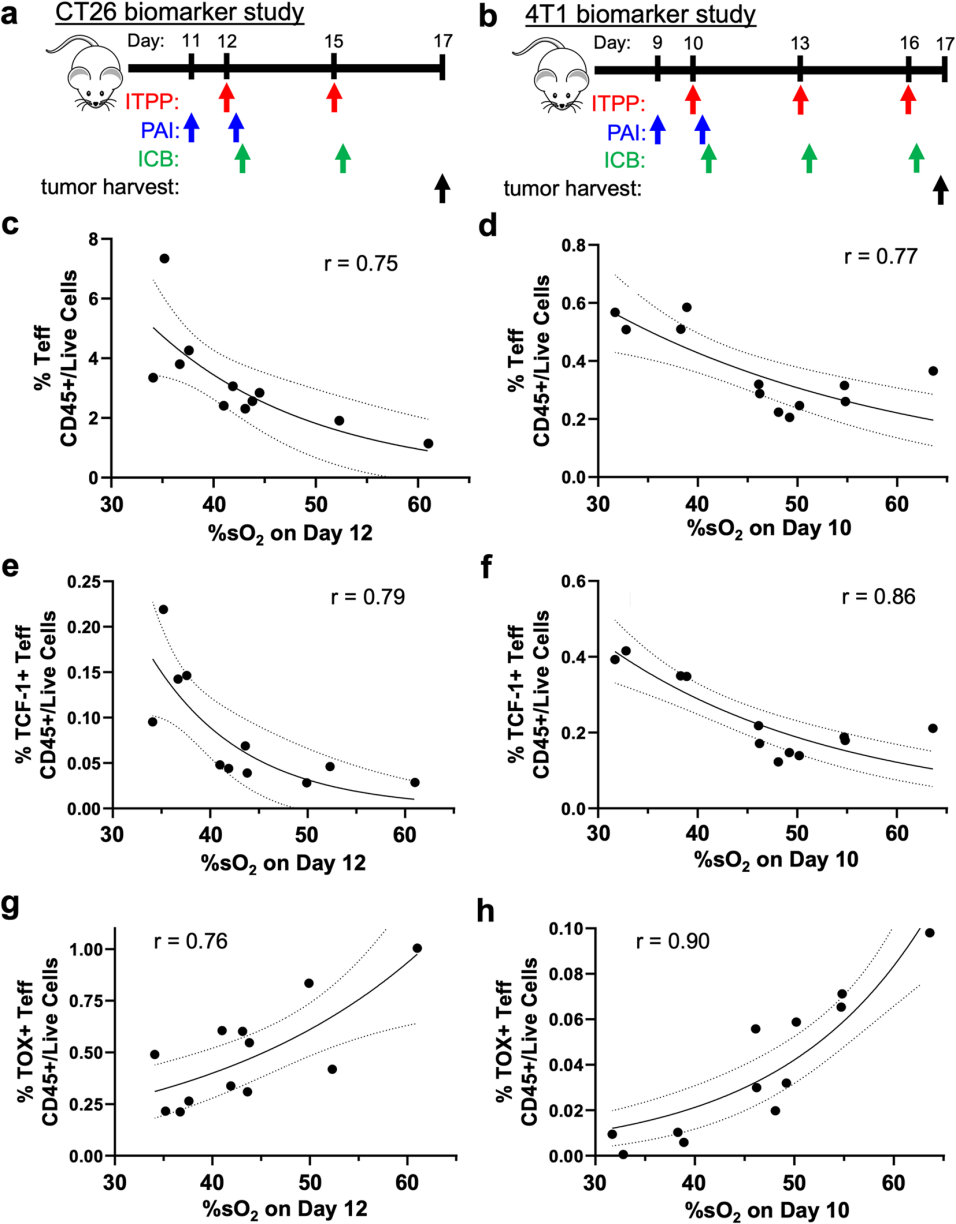
%sO_2_ after the first ITPP treatment correlated with intratumoral frequencies of Teffs following ITPP and ICB treatment. (**a**,**b**) The study timelines. PAI was performed 3 h after administering ITPP. ICB was administered immediately after PAI on the initial treatment day. ICB was administered 3 h after ITPP on days without PAI. Negative correlations between %sO_2_ three hours after ITPP and the frequencies of (**c**) CD8 and CD4 + FoxP3- T cells in CT26 tumors, (**d**) CD8 and CD4 + FoxP3- 4T1 tumors, (**e**) TCF-1 + CD8 and TCF-1 + CD4 + FoxP3- T cells in CT26 tumors, and (**f**) TCF-1 + cD8 and TCF-1 + CD4 + FoxP3- T cells in 4T1 tumors after two or three commination ITPP, αCTLA-4 and αPD-1 treatments. Positive correlations between %sO_2_ three hours after ITPP and the frequencies of TOX + CD8 and TOX + CD4 + FoxP3- T cells in (**g**) CT26 and (**h**) 4T1 tumors after ITPP, αCTLA-4 and αPD-1 treatments. Dotted lines represent the 95% confidence interval while the solid lines represent the linear best fit line. *n* = 11–12*.*

As the previous experiment established post-ITPP %sO_2_ as an early response biomarker for tumor control in both CT26 and 4T1 tumors in our studies, we sought to determine the cellular mechanism driving the ability of %sO_2_ to be related to tumor control by ITPP and ICB. We analyzed the immune infiltrate in CT26 and 4T1 tumors after PAI, and compared our immunophenotyping results to post-ITPP %sO_2_ to determine the cellular changes associated with increased oxygen unloading by RBCs in the tumor. The timeline of this study for both tumor models is shown in Fig. 8a,b, which are the same timelines as shown in Fig. 7a,b.

We observed specific correlations when comparing the frequencies of certain immune cell populations in the tumors to post-ITPP %sO_2_. Teff frequencies in CT26 and 4T1 tumors negatively correlated with their respective post-ITPP %sO_2_ measurements (Fig. 8c and d). These Teff populations were not associated with increased cytotoxic function, however, as the examination of granzyme B expression in Teff cells did not yield a correlation between granzyme B-expressing Teff frequencies and post-ITPP %sO_2_ (data not shown). Among the Teff populations, we observed a negative correlation between the frequencies of TCF-1 + Teff and their respective post-ITPP %sO_2_ in both CT26 and 4T1 tumors (Fig. 8e and f). Furthermore, TOX + Teff frequencies had a positive correlation with post-ITPP %sO_2_ in CT26 and 4T1 tumors (Fig. 8g and h). After assessing the frequencies of different myeloid cell populations as well as the frequencies of stimulatory and inhibitory marker-expressing myeloid populations, there were no significant correlations between any myeloid cell populations and post-ITPP %sO_2_ (data not shown).

As TCF-1 is a marker of fit, progenitor T cells^57^ whereas TOX is a marker of exhausted T cells^58^, our data indicates that decreased %sO_2_ after ITPP is associated with less Teff exhaustion in both CT26 and 4T1 models. Because post-ITPP %sO_2_ positively correlates with tumor mass, increased Teffs, TCF-1 + Teffs, and decreased TOX + Teffs are also associated with low tumor mass following ITPP and ICB treatment. While these Teff populations are not more cytolytic, a more pronounced infiltration of these T cells can increase their antitumor effect by sheer volume. Therefore, more TCF-1 + Teffs and fewer TOX + Teffs in tumors mediate the tumor control induced by ITPP, αCTLA-4 and αPD-1. Low post-ITPP %sO_2_ can therefore be an early response biomarker in our studies of combination ITPP and αCTLA-4 and αPD-1 efficacy as well as an indicator for increased progenitor Teff infiltration.

## Discussion

Our work showed that ITPP increases oxygen unloading from hemoglobin, leading to increased tumor immunogenicity and synergy with ICB, and was related to tumor control by combination ITPP and ICB in the CT26 and 4T1 tumor models. Low %sO_2_ induced by ITPP promotes the conversion of CT26 and 4T1 into “hot” tumors by increasing the frequencies of Teffs, while decreasing the frequencies of monocytes in the tumors. Combination treatment of ITPP, αCTLA-4 and αPD-1 significantly delayed tumor growth in both CT26 and 4T1 models in a manner related to low post-ITPP %sO_2_. Lastly, low post-ITPP %sO_2_ was associated with increased frequencies of Teffs, as well as TCF-1 + Teffs, and associated with decreased frequencies of TOX + Teffs. Teff infiltration as well as progenitor, non-exhausted Teff subsets contributed to the antitumor effect of combination therapy ITPP, αCTLA-4 and αPD-1.

Both tumor models had a similar modulation of the immune cell composition in response to ITPP. Both CT26 and 4T1 tumors increased their frequencies of Teff cells at low %sO_2_ induced by ITPP (Fig. 2b,c, 5b,c), which carried over to the response to combination ITPP, αCTLA-4 and αPD-1 (Fig. 8c,d). Our results with our models of breast and colon cancers are similar to previous studies that showed elevated immune cell influx into tumors of rat and mouse models of head and neck, lung, colon, and pancreatic cancers^34–36^. However, more studies with additional tumor models are needed to investigate whether the conversion of immunogenically “cold” tumors into “hot” tumors that have high T cell penetrance is a common feature of all cancer types^1,25^. In particular, while CT26 and 4T1 are considered to be moderately hypoxic tumors^59,60^, both normoxic and highly hypoxic tumor models should be considered. Most importantly, we have performed the first study that combines ITPP with ICB. Additional pre-clinical studies that use ITPP to convert “cold” tumors into “hot” tumors should also include ICB treatment.

Our results showed that ITPP only produced a significant change in %sO_2_ three hours after the first treatment, but not after the second and third treatments (Figs. 1c and 4c). This result showed the value of in vivo imaging to monitor longitudinal changes in tumor models, especially because we assumed that a sustained change in %sO_2_ would occur after each ITPP treatment. Future studies can evaluate other longitudinal doses and timings of ITPP and ICB that may elucidate the longitudinal pharmacodynamics of combination treatments. Other agents that promote oxygenation in the TME can also be studied using longitudinal PAI studies. For example, PAI has been used to detect the release of O_2_ from hemoglobin by an analog of sulfoquinovosylacylglycerol^61^ and also detected the production of O_2_ by metallic nanoparticles^62,63^. Future studies could also use PAI to image an untreated control group or a group only treated with ICB, although no change in %sO_2_ would be expected.

Our study indicates that further technical improvements are warranted for evaluating tumor %sO_2_ with PAI. For example, the mechanism of ITPP should unload oxygen in more hypoxic tumor regions and reduce the heterogeneity of oxygen distribution within the tumors. We only analyzed the average tumor %sO_2_ due to PAI measurement imprecision, rather than analyzing pixelwise values of %sO_2_. Also, our methodology only analyzed one imaging slice through the tumor. A more precise imaging method and/or multislice imaging to analyze the entire tumor may provide opportunities to evaluate %sO_2_ heterogeneity as a complementary biomarker [e.g., replacing %sO_2_ with a ‘%sO_2_ heterogeneity’ biomarker in Figs. 2, 5, 7, and 8). We did not evaluate total hemoglobin (HbT) in our study despite measuring Hb and HbO_2_ (where HbT = Hb + HbO_2_) because light scattering and absorption in tissues can influence HbT measurements at different tissue depths^64^. The ratiometric measurement of %sO_2_ mitigates this effect, making %sO_2_ a more reliable parameter^65^. Yet, accounting for light scattering and absorption in future PAI studies may lead to evaluations of HbT and may also improve the precision of %sO_2_ measurements^66^.

We used %sO_2_ measurements with PAI to verify the mechanism of ITPP-mediated release of O_2_ from HbO_2_ in vivo. However, PAI cannot directly measure oxygenation in the TME, which is important for studies of immunotherapy studies because immune cells interact with tumor cells within the TME. For comparison, EPR imaging and MRI can directly evaluate pO_2_ in the TME and have been used to confirm that ITPP increases pO_2_ in the TME^31–33^. However, EPR imaging and MRI cannot confirm the mechanism of ITPP-mediated release of O_2_ from HbO_2_. Therefore, the combination of these previous reports and our study strengthen our understanding of how ITPP potentiates ICB.

Oxygenation of the TME can also be improved by increasing tumor vascular perfusion^67^. Previous studies showed the capacity of ITPP to normalize vasculature and promote tissue perfusion^68^. We have invented a method that can quantitatively measure the tumor vascular perfusion rates in tumor models, known as Dynamic Contrast Enhanced (DCE) PAI^69^, which can be performed with %sO_2_ PAI during a single scan session^70^. Future studies can use a combination of %sO_2_ PAI and DCE PAI for pre-clinical investigations of ITPP with ICB. For comparison, EPR imaging, bioluminescence imaging, PET and MRI have also been used to evaluate the tumor response to ITPP^31–33,35,47^. However, EPR imaging and bioluminescence imaging cannot measure vascular perfusion, and DCE PET is rarely employed due to poor spatial resolution. DCE MRI has many technical limitations^71–73^ and is often relegated to qualitative contrast-enhanced MRI that cannot measure vascular perfusion rates^74,75^. Therefore, the combined methodology of %sO_2_ PAI and DCE PAI is a new paradigm for imaging the effects anti-cancer therapies in pre-clinical tumor models. Furthermore, %sO_2_ PAI is now approved for imaging breast cancer^76^, and we are translating DCE PAI to clinical practice. A combined protocol for %sO_2_ PAI and DCE PAI may eventually benefit clinical treatment studies, and may aid in stratifying patients into treatment groups that would or would not benefit from combination treatments that improve tumor oxygenation prior to ICB.

## Methods

### ITPP

We synthesized ITPP with a Na^+^ counterion based on a published procedure[78,79]. The sodium salt of phytic acid (Themofisher, Inc.) was acidified by Dowex H^+^ resin (Sigma–Aldrich). Triethylamine was used to protect the acid. 1,3-dicyclohexylcarbodiimide (8 eq, Sigma–Aldrich) dissolved in acetonitrile was added to the water solution of the triethylamine protected compound and refluxed overnight. After dilution with ddH_2_O, the solid formed was vacuum filtered away. The pass-through liquid was lyophilized to dryness. Dowex Na form resin (Sigma–Aldrich) was used to ionize the dissolved water solution from the previous step to sodium form. The reaction crude was purified by HPLC (1% to 30% acetonitrile in water gradient). 1H NMR (D2O, 600 MHz): 4.43 (s, 6H). C NMR (D2O, 600 MHz): 75.64 (s): ‘P NMR (D2O, 300 MHz): − 9.2536. (ESI): *m/z* Calcd for C6H12O21P6 [M^+^]: 605.98, Found: 607.03. [M^−^]: 604.98, Found:604.85.

To prepare ITPP for in vivo studies described below, ITPP was dissolved in sterile phosphate-buffered saline (PBS) for a final concentration of 22.22 g/mL. The ITPP solution was then passed through a 0.22 μm filter followed by processing using an endotoxin removal kit (Thermo Scientific, 88276). ITPP was injected intraperitoneally at a dose of 2.222 g/kg.

### Antibodies

To prepare antibodies for in vivo studies described below, the αCTLA-4 (9H10) and αPD-1 (RMP1-14) antibodies (Leinco Technologies, C2841 and P372, respectively) were diluted in PBS and injected intraperitoneally at doses of 5 mg/kg and 12.5 mg/kg, respectively. PBS was injected intraperitoneally as the vehicle control.

### Cell lines

To prepare tumor models for in vivo studies described below, CT26 murine colon carcinoma cells (ATCC, CRL-2638) were grown in Roswell Park Memorial Institute 1640 (RPMI) media supplemented with 10% FBS, 2% penicillin and streptomycin, 1% L-glutamine, and 1% sodium pyruvate in tissue culture-treated flasks. 4T1 murine mammary epithelial cells (CVCL_0125, ATCC, CRL-2539) derived from female mice were also grown in tissue culture-treated flasks supplemented with RPMI and additional 10% FBS, 1% penicillin and streptomycin, and 1% L-glutamate, but lacking HEPES and sodium pyruvate. No testing was done on these cell lines because the cell bank ATCC uses the Promega PowerPlex 18D System to identify cell lines by Short Tandem Repeat analyses. Cells were manipulated upon reaching 90% confluence prior to twenty passages. CT26 and 4T1 tumor cell lines were initially selected due to their sensitivity to combination αPD-1 and αCTLA-4 treatment without complete tumor rejection^52–56^.

### Pre-clinical tumor models and treatments

All experiments involving mice were performed in accordance with the relevant guidelines and regulations and were conducted according to the protocol number 00001779-RN01 approved by the Institutional Animal Care and Use Committee at the UT MD Anderson Cancer Center. Furthermore, all studies with mice were performed in accordance with the 10 essential items of the ARRIVE guidelines. Female BALB/cJ (IMSR_JAX:000651) mice were purchased from the Jackson Laboratory and housed in a pathogen-free facility fully accredited by the Association for Assessment and Accreditation of Laboratory Animal Care. Mice were initially 6–8 weeks old and approximately 20 g in weight. To develop the CT26 model, 1 million cells were resuspended in 200 µL PBS injected intradermally into the right flank. To develop the 4T1 model, 40,000 cells were resuspended in 50 µL PBS and injected into the fat pad of the 4th inguinal nipple. All cells were implanted in mice between the ages of 5–8 weeks and at least 20 g in weight. Tumor volumes were approximated by measuring the longest diameter (a) and the orthogonal diameter (b) using digital calipers and calculating a^2^b/2. Mice were randomly selected for each cohort. Each mouse was humanely euthanized using CO_2_ inhalation or isoflurane anesthetic overdose followed by cervical dislocation as a secondary method.

Treatment with ITPP began when CT26 tumors reached 5–6 mm in diameter at Day 12, and when 4T1 tumors reached 3–4 mm in diameter on Day 10. Mice were excluded from the study if their tumors were not visible or exceeded 200 mm^3^ upon blind distribution prior to the first treatment. The timelines of our %sO_2_ PAI studies (Figs. 1 and 4), immunogenicity studies (Figs. 2 and 5), tumor growth and survival studies (Figs. 3 and 6), and biomarker studies (Figs. 7 and 8) are shown in each figure.

For PAI studies, 10 mice of each tumor model were used, with 5 mice tested with ITPP and 5 tested with control. One mouse with a CT26 tumor in the ITPP treatment group expired prior to the first imaging scan (Fig. 1). Three mice with a 4T1 tumor expired before the last imaging scan (Fig. 4). A total of 20 mice were used for immunogenicity studies. For tumor growth and survival evaluations, 60 mice of each tumor model were used, randomly distributed among four treatment tests that were conducted in triplicate, with 5 mice per group. In addition, 26 mice were used for biomarker experiments. Sample sizes ranged from 4 to 12 mice, with tumor growth kinetics and survival studies conducted in triplicate. The range of sample sizes reflected occasional sample losses or unreliable results during biomarker studies that were excluded from the final analysis which ensures that the reported results are rigorous.

### Photoacoustic imaging

To perform PAI, a mouse was first anesthetized with 2–5% isoflurane in 21% oxygen breathing gas. The mouse was then secured to a cradle with a snorkel nosecone and placed in an inVision instrument (iThera Medical GmbH, inVision 256-TF), immersed in a tank containing deionized water (Millipore Milli-Q Ultrapure water system, Millipore Sigma, Burlington, MA) pre-heated to 36 °C. This bath temperature allowed anesthetized mice to maintain a body temperature of 37 °C as monitored with a rectal temperature sensor (SA Instruments, Inc., Stony Brook, MA) in preliminary imaging feasibility studies prior to treatment studies. The mouse was allowed to equilibrate to temperature and establish a consistent breathing rate for 12 min, which contributed to consistently high image quality. During this equilibration period, an anatomical ultrasound localizer scan was acquired with 31 contiguous image slices, each with 1 mm thickness, collected around the expected location of the tumor using 800 nm absorbance and 5 averages, which took ~ 4 min to acquire.

To evaluate %sO_2_, PA images were acquired for 1 slice centered on the tumor. A B-mode ultrasound image was acquired, which was used to identify the region-of-interest that represented the tumor in the image sets. Using an OPO laser in the inVision instrument to produce a surface fluence of 20 mJ/cm^2^, we acquired images with 5 wavelengths at 700, 730, 760, 800, and 850 nm, using 6 averages at each wavelength, and repeated 40 times at 10 Hz laser repetition rate, for a scan time of 2.0 min. Each image was reconstructed using a back-projection method, and each image set was spectrally unmixed via linear regression to generate parametric maps of oxy- and deoxy-hemoglobin, which were used to produce maps of %sO_2,_ using viewMSOT v 4.0 (iThera Medical). The %sO_2_ values of the pixels were averaged for all tumor pixels within the region of interest representing the tumor, where the tumor region was identified in the B-mode ultrasound images. The temporal profiles of each biomarker were evaluated to ensure that results were consistent over the 2-min acquisition, and the average over all time points was determined for each biomarker_._

### Ex vivo tumor cell analysis

Tumors harvested for flow cytometry were minced and digested for 30 min at 37 °C in RPMI supplemented with Collagenase H (Sigma-Aldrich, 11074059001) and DNAse (Roche, 4716728001). After digestion, tumor samples were mashed against 70 µm cell strainers (Corning Life Sciences, 352350) using the textured end of a 1 mL syringe plunger. Once a single cell solution was produced, the samples were normalized by cell count before being stained with an extracellular antibody cocktail and a UV viability dye (Invitrogen, L34961). Cells were then fixed using a fixation buffer (eBioscience, 00-5523-00). After fixing cells, they were stained intracellularly according to the instructions provided with each antibody. Antibodies included CD45 (BD Biosciences, 748371), CD4 (BioLegend, 100548), CD8 (BioLegend, 100734), Ly6G (BD Biosciences, 563979), F4/80 (BioLegend, 123141), CD11c (BioLegend, 117306), CD11b (BioLegend, 101254), Ly6C (BioLegend, 128026), FoxP3 (BioLegend, 320008), TOX (eBioscience, 12-6502-8), TCF-1 (BD Biosciences, 566693). Flow cytometry was performed with the gating strategy used in Reference 9.

### Statistics

Tumor growth and survival experiments used three biological repeats for data analysis. Oxygenation results are presented as mean ± standard deviation, while tumor growth results are presented as mean ± standard error of the mean. All statistical analyses were conducted through GraphPad Prism 9.0.0. The *p*-values and significance were determined by one-way ANOVA. Correlation studies were analyzed using Linear and Exponential Regressions to determine goodness of fit and 95% confidence intervals. No blinding or masking of the identity of the samples or mice was performed during the analyses.

## Data Availability

All data is available upon request by contacting the corresponding author.
